# Gender disparities in eHealth literacy among Chinese university students and their decomposition

**DOI:** 10.3389/fpubh.2026.1919426

**Published:** 2026-09-03

**Authors:** Yuyao Song, Yusupujiang Tuersun, Qingping Zhou, Wenyu Wang, Yao Yu, Chenxi Wang, Hanyue Liu, Yao Wu, Yuying Xie, Zhenning Liang, Yi Qian

**Affiliations:** 1 School of Health Management, Southern Medical University, Guangzhou, China; 2 Lianyungang Market Supervision Administration, Lianyungang, China; 3 Shenzhen Maternity and Child Healthcare Hospital, Women and Children’s Medical Center, Southern Medical University, Shenzhen, China; 4 School of Public Health, Southern Medical University, Guangzhou, China; 5 The First Affiliated Hospital of Guangzhou Medical University, Guangzhou, China; 6 Stomatological Hospital of Southern Medical University, Guangzhou, China; 7 Shenzhen Longhua Maternity and Child Healthcare Hospital, Shenzhen, China; 8 Department of Academic Affairs, The Seventh Affiliated Hospital of Sun Yat-sen University, Shenzhen, China

**Keywords:** digital health, eHealth literacy, Fairlie decomposition model, gender disparities, health equity, university students

## Abstract

**Background:**

As a high-frequency user group of the internet, university students’ eHealth literacy not only affects their own health but also has a radiating effect on the health literacy of the entire population. Gender disparities may lead to an imbalance in eHealth literacy among university students, thereby affecting their health status. Understanding these disparities is important from a public health perspective for designing equitable digital health interventions.

**Methods:**

The eHealth Literacy Scale was used to assess the eHealth literacy levels of 7,230 university students from various institutions and majors across 10 regions. Chi-square tests and binary logistic regression were employed to analyze factors associated with gender disparities in eHealth literacy, and the Fairlie decomposition model was used to quantify the contribution of each factor.

**Results:**

The average eHealth literacy score of the sampled Chinese university students was 29.22 ± 6.68. Of the 7,230 students, 4,135 (57.2%) scored below the 32-point eHEALS cut-off. Regarding gender, female students had a significantly higher rate of inadequate eHealth literacy (2,847/4,820, 59.07%) compared with male students (1,288/2,410, 53.44%; *p* < 0.001). The Fairlie decomposition analysis showed that 55.0% of the disparity in eHealth literacy was attributable to unmeasured variables, differences in coefficients, measurement error, or residual confounding, while 45.0% resulted from observed factors. Among these, depressive symptoms (PHQ-9) were the largest contributing factor (28.57%), followed by type of household (14.29%), monthly per capita household income (10.71%), and exercise level (−3.57%).

**Conclusion:**

Significant gender disparities exist in eHealth literacy among Chinese university students, with male students showing higher levels. This disparity is associated with mental health status, socioeconomic status, and lifestyle behaviors. This study proposes several potential strategies including fostering a gender-equal atmosphere, optimizing digital resource allocation, and strengthening campus mental health services that may help to narrow the gap.

## 1 Introduction

Health literacy refers to an individual’s ability to obtain, process, and understand basic health information and services needed to make appropriate health decisions (1), and it is a determinant of health outcomes. Improving the health literacy of the entire population is one of the most fundamental, cost-effective, and efficient measures to enhance national health levels. In the context of the “Healthy China” strategy and the deep integration of digital technologies, the internet has become a primary channel for the public to access health information and services. However, issues such as information overload and variable quality online have also emerged (2), placing higher demands on individuals’ abilities to effectively search for, discern, understand, and apply online health information. Against this backdrop, the importance of electronic health (eHealth) literacy has become even more pronounced.

In May 2005, the World Health Organization (WHO) defined eHealth at the 58th World Health Assembly as “the transfer of health resources and healthcare information by electronic means, enabling health professionals and users to disseminate and access health information” (3). Building on this, Norman and Skinner first defined eHealth literacy in 2006 as “the ability to seek, find, understand, and appraise health information from electronic sources and apply the knowledge gained to addressing or solving a health problem” (4). Research indicates that a high level of eHealth literacy not only promotes individuals’ effective use of health information but also helps improve their health knowledge, fostering positive health behaviors and lifestyles (5–7). It can also enhance the efficiency and quality of doctor-patient communication, thereby increasing the effectiveness and satisfaction of healthcare services (8).

From a public health perspective, understanding gender disparities in eHealth literacy is critical for achieving health equity. Gender inequalities in health reflect systemic disparities in health outcomes and access to resources based on gender. Digital health technologies offer promising solutions to address these disparities, but their effectiveness depends on users’ eHealth literacy (9). Some studies have shown that gender gaps in digital access and skills may exacerbate existing health inequalities, especially in low- and middle-income countries (10, 11). For young adults, these gaps may become entrenched over time, leading to lifelong disparities in health-seeking behaviors and health outcomes. Therefore, identifying and addressing gender gaps in eHealth literacy is a prerequisite for designing equitable digital health interventions.

University students constitute the backbone and crucial reserve talent for national and societal development. Their health literacy and physical and mental well-being significantly impact the country’s future development. University students process information quickly, have high educational levels, and exhibit a clear radiating effect on their families (12). As a primary active group on the internet, most university students rely on the internet as their main tool for receiving health information and making health-related decisions (13). On one hand, university students are in a critical period for forming health behaviors and concepts (14). Possessing good eHealth literacy can help them cope with common health issues and risks during young adulthood, such as staying up late, imbalanced diets, and psychological stress (15). Empirical studies show that individuals with higher eHealth literacy tend to have healthier lifestyles (16). Other research suggests that eHealth literacy is a protective factor against anxiety and depressive symptoms; individuals with high eHealth literacy can utilize online resources more effectively for psychological adjustment, demonstrating better psychological resilience (17). On the other hand, despite their frequent internet use, university students often exhibit significant shortcomings in evaluating the quality of online information and applying it. Their ability to acquire and identify correct online health information is insufficient, and they struggle to integrate the obtained health information into their own behaviors and lifestyles (18, 19). Research indicates that while university students are generally familiar with and willing to use the internet for health information, their overall eHealth literacy levels tend to be low, particularly their capacity for critically evaluating the credibility and quality of information, which often fails to meet required standards (12). A study on university students’ eHealth literacy during the early COVID-19 pandemic found that although most could frequently access health information online, a considerable proportion had difficulty distinguishing commercial advertisements from evidence-based scientific health content (20). Another study discovered that even when young people acquire health-related knowledge, they often fail to translate it into sustained health behaviors (18).

University students are in a critical transition phase from family-monitored health to self-managed and family health responsibility. They play a pivotal role in the health information dissemination chain. Studies show that university students not only seek health information for themselves but also frequently search for and disseminate health information for family and friends, acting as important health information intermediaries within their social networks (21). Therefore, enhancing the eHealth literacy of university students not only positively impacts their own health but is also significant for comprehensively improving the health literacy of the population in the future.

In 2023, the total enrollment in higher education in China reached 47.63 million. University students, as one of the high-frequency user groups of the internet, are receiving growing attention with regard to their eHealth literacy. From a policy perspective, enhancing eHealth literacy has become a vital component of the national health strategy. The “Outline of the Healthy China 2030 Plan” explicitly lists the “National Health Literacy Promotion Initiative” as a core task, proposing to “improve the public’s ability to use the internet to access health information.” As future disseminators of health concepts, university students have eHealth literacy levels that directly shape the implementation effectiveness of the strategy. The WHO’s “Global Strategy on Digital Health 2020–2025” also emphasizes that building digital health capacity among youth groups is key to reducing health inequalities, further highlighting the policy relevance of research on university students’ eHealth literacy (22). At the research level, with the rapid development of the internet in the healthcare field, an increasing number of researchers are exploring eHealth literacy. A review showed that the eHealth literacy of Chinese university students is generally at a low-to-medium level, influenced by multi-dimensional factors including individual characteristics, interpersonal relationships and policies (23). A large-scale national survey found that students’ average eHealth literacy score was below the recognized passing mark, indicating insufficient ability to effectively navigate the complex online health information environment (24). Regarding influencing factors, research confirms that senior students, those majoring in medicine-related fields, and students reporting good health status typically exhibit higher eHealth literacy levels. Furthermore, socioeconomic factors play a significant role; students from urban areas (25, 26), with highly educated parents and higher family income levels (27), show significant advantages in accessing and utilizing digital health resources. Existing research also reveals the intrinsic link between mental health and eHealth literacy, with depressive and anxious symptoms confirmed as significant negative predictors of eHealth literacy (28). Simultaneously, most scholars believe that healthy behavioral habits and eHealth literacy are mutually reinforcing (25, 29), jointly contributing to a healthy lifestyle.

Existing research has identified several factors associated with eHealth literacy among university students. These include demographic characteristics, socioeconomic status, lifestyle behaviors, and mental health status. However, most of these studies have treated gender merely as a control variable, reporting mean score comparisons without systematically decomposing the sources of gender disparities. Specifically, while previous research has identified correlates such as socioeconomic status, mental health, and lifestyle behaviors, it remains unclear which of these measured factors account for the gender gap and to what extent unexplained factors play a role. This lack of decomposition-focused analysis limits the development of targeted, gender-sensitive interventions, as evidence-based strategies require not only knowledge that a gap exists but also an understanding of its specific drivers. Therefore, in addition to examining gender disparities in eHealth literacy levels, there is an urgent need to quantitatively decompose the sources of these disparities to inform precision health promotion and health equity-oriented policy making.

Considering these factors, this study aims to: (1) assess the current status of eHealth literacy among Chinese university students and examine whether gender disparities exist; (2) analyze factors associated with gender disparities in eHealth literacy for male and female university students separately; (3) utilize the Fairlie decomposition model to quantify the contributions of observable factors to the gender gap. The findings will provide a new perspective for understanding formation mechanisms and offer an empirical basis for developing future gender-specific health literacy intervention strategies.

## 2 Methods

### 2.1 Data source

This study is a cross-sectional survey. Between January and February 2023, researchers distributed anonymous electronic questionnaires via the Wenjuanxing platform^1^ using a convenience sampling method. The recipients of the questionnaires were university students from 10 regions, including Guangdong Province, Shanghai, and Jiangsu Province, covering multiple fields of study such as economics, medicine, management, and literature. The 10 regions were selected based on the distribution of cooperative universities and survey accessibility, and were chosen to cover eastern, central and western regions of China as far as possible to increase the diversity of the sample. The sample size of this study is large, with a wide coverage and encompassing multiple disciplines, providing a sufficient data foundation for analyzing gender disparities and their influencing factors. Due to the convenience sampling design adopted in this study, caution should be exercised when generalizing the research results. This study was approved by the Biomedical Ethics Committee of Southern Medical University, specifically document No. 46 from the Ethics Review Committee (2023). Before the survey began, all participants signed informed consent forms. The distribution, collection of questionnaires, and data storage were all conducted through the Wenjuanxing platform. This study strictly adheres to all applicable laws and regulations. In public reports, strict measures are taken to protect the privacy of participants and ensure that no personally identifiable information is disclosed. After data collection, the dataset was exported and converted to EpiData format (Version 3.1, The EpiData Association, Odense, Denmark) for subsequent statistical analysis.

**Figure 1:**
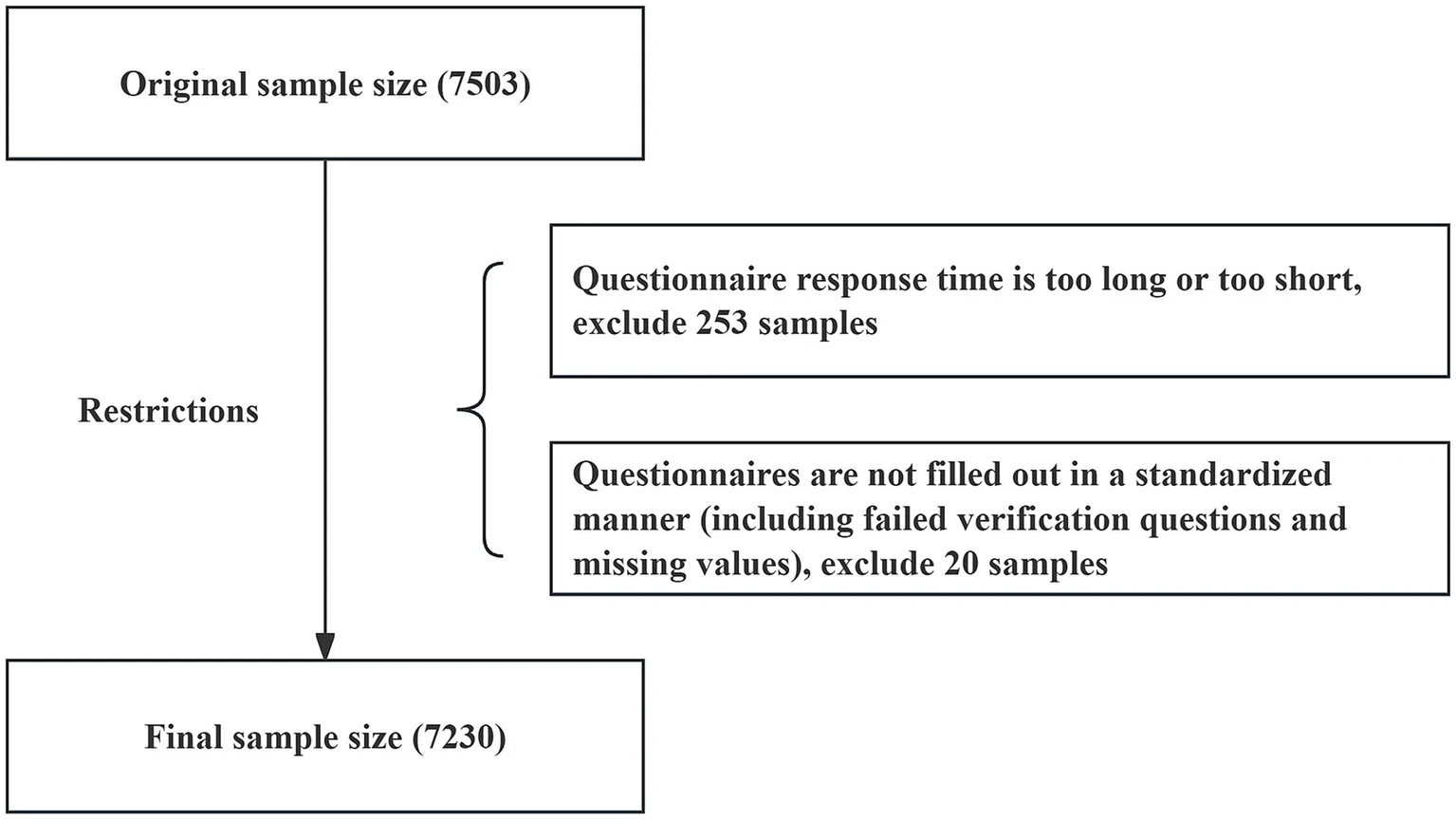
Participant screening flowchart.

To ensure the accuracy and reliability of the data and minimize careless and invalid responses, the questionnaire included two verification questions: one review question, and one basic knowledge question (where is the capital of China). If any respondent answered incorrectly to these questions, the questionnaire would be deemed invalid and excluded from subsequent analysis. Response times shorter than 90 s (indicating careless and random responses) or longer than 1800 s (indicating interrupted or distracted submission) were defined as abnormal and removed. A total of 7,503 questionnaires were collected for this study. After two-step quality control screening, 253 questionnaires with abnormal response times were first removed, followed by the exclusion of 20 invalid questionnaires (including those with incorrect answers to verification questions, incomplete responses, and obvious logical inconsistencies). All respondents who failed the verification questions were covered in the above two exclusion groups. Finally, 7,230 valid questionnaires were retained for analysis, with an effective recovery rate of 96.4%. Participants in this study must meet the following criteria: they must be undergraduate students; have normal cognitive function and be able to complete the questionnaire independently. Exclusion criteria include incorrect answers to the verification questions. The exclusion process is shown in Figure 1.

### 2.2 eHealth literacy

This study employed the Chinese version of the eHEALS to measure the eHealth literacy levels of university students. The eHEALS was developed by Norman and Skinner in 2006, based on the concept of eHealth literacy and the Lily’s Model, aiming to assess an individual’s ability to search for, filter, understand, evaluate, and utilize health information (4). Since its introduction, the eHEALS has become the most commonly used tool for evaluating eHealth literacy across different populations (30). Multiple studies targeting various groups have shown that the Cronbach’s α coefficient of the eHEALS is relatively high, generally ranging from 0.84 to 0.94. This indicates that the scale demonstrates high internal consistency and reliability. The scale consists of eight items, covering three categories of skills related to the use of online health information and services: application ability (items 1–5), judgment ability (items 6–7), and decision-making ability (item 8). The scale uses a five-point Likert scale for scoring, with a range from one to five. The total score for the eight items ranges from 8 to 40 points, with higher scores indicating higher levels of eHealth literacy.

For the convenience of population stratification analysis and public health intervention reference, this study adopted the 32-point cut-off which is widely used in existing research for grouping (31–33). This threshold corresponds to an average item score of 4 on the 5-point Likert scale, meaning that respondents generally have relatively sufficient ability to search for, critically evaluate, and apply online health information to solve health problems. For Chinese populations, multiple validation and application studies have also taken 32 points as the reference threshold for evaluating eHealth literacy level (26, 32, 34, 35), including investigations targeting Chinese university students, which supports its applicability in this study population. Therefore, in the subsequent analysis, this study adopted the widely recognized cut-off score of 32 points to classify the eHealth literacy of male and female university students into two categories: above the cut-off (referred to as “qualified” for brevity in this study, ≥32 points) and below the cut-off (referred to as “unqualified” for brevity, <32 points) (36, 37). It should be noted that this value serves as a commonly used reference threshold rather than an absolutely validated gold standard. The overall Cronbach’s alpha coefficient for this study was 0.974, with 0.979 for the male university student group and 0.970 for the female university student group. The coefficients are slightly higher than the range typically reported in previous related studies, which can be attributed to the high homogeneity of the university student sample in terms of age, educational level and digital exposure. For a unidimensional scale like the eHEALS, higher internal consistency in a homogeneous young adult sample is consistent with psychometric expectations and indicates good reliability of the scale in this study population.

### 2.3 Grouping variables

According to the gender types reported by respondents in the survey, respondents were divided into male and female.

### 2.4 Covariates

To ensure the theoretical adequacy of the analytical framework and sufficient control for confounding, we selected candidate covariates based on systematic reviews and classical empirical studies on the influencing factors of eHealth literacy (12, 23, 25). The covariates covered four well-recognized domains: demographic characteristics, sociological characteristics, personal lifestyle behaviors, and mental health status, all of which have been widely verified to be associated with eHealth literacy levels in university student populations.

### 2.5 Demographic characteristics

The participants were classified according to two variables: type of household (rural or urban) and personal religious belief (yes or no).

### 2.6 Sociological characteristics

The respondents were classified into four groups based on their academic performance, determined by their class ranking: below 25, 25–50%, 50–75%, and above 75%. Furthermore, monthly per capita household income was divided into four categories: less than 2,500 yuan, 2,500–5,000 yuan, 5,000–10,000 yuan, and above 10,000 yuan.

### 2.7 Personal lifestyle

The variables of exercise, smoking, and drinking were dichotomized into a binary format, with responses of “yes” or “no” assigned to each item. The term “exercise” was defined as engaging in physical activity on three or more occasions over the past month, with each session lasting at least 30 min. Moreover, the use of tobacco products and alcoholic beverages in the past month was recorded as a dichotomous variable (yes or no).

### 2.8 Mental health status

The 7-item Generalized Anxiety Disorder scale (GAD-7) was employed to assess the prevalence and severity of anxiety symptoms among the study participants. The tool comprises seven items, each rated on a 4-point scale ranging from 0 to 3. The rating scale is as follows: 0 = “not at all,” 1 = “a few days,” 2 = “more than half the time,” and 3 = “almost every day.” A score below 5 indicates the absence of anxiety symptoms, while a score of 5 or higher indicates the presence of anxiety symptoms (38). In this study, the GAD-7 exhibited a Cronbach’s alpha coefficient of 0.944.

The 9-item Patient Health Questionnaire (PHQ-9) was employed to assess depressive symptoms among the participants. The PHQ-9 is comprised of nine items and employs the same scoring method as the GAD-7. A score below 5 indicates the absence of depressive symptoms, whereas a score of 5 or higher suggests the presence of depressive symptoms (39). In this study, the PHQ-9 exhibited a Cronbach’s alpha coefficient of 0.931.

### 2.9 Statistical analysis

The fundamental demographic characteristics and lifestyles of the study population were analyzed in a descriptive manner. Continuous variables were described by the mean ± standard deviation, while categorical variables were described by the percentage. A chi-square test was employed to analyze the distribution of eHealth literacy levels among male and female university students. Subsequently, a binary logistic regression model was constructed in this study to identify the main factors associated with the level of eHealth literacy among male and female university students. The outcome variable was a binary categorization of eHealth literacy status, coded as 1 for “qualified” (eHEALS total score ≥32) and 0 for “unqualified” (eHEALS total score <32). The independent variables were selected from four domains: demographic characteristics, socioeconomic characteristics, personal lifestyle, and mental health status. For all categorical independent variables, the group with stronger baseline reference significance was set as the reference category.

For the binary logistic regression analysis, we first performed univariate analysis to preliminarily explore the association between each covariate and eHealth literacy level. Variables with *p* < 0.05 in univariate analysis, as well as core variables that have important theoretical significance even if they are not statistically significant in the univariate test, were included in the multivariable model based on professional judgment. Statistically significant predictors (*p* < 0.05) were retained in the final multivariable model after stepwise adjustment. The aforementioned statistical analyses were conducted using IBM SPSS Statistics 23 software.

For the Fairlie decomposition analysis (FDA), all candidate covariates from the four domains were entered into the model, rather than only the variables that were statistically significant in the regression. This specification minimizes omitted variable bias and allows for a more accurate decomposition of the explained and unexplained components of the gender disparity. The decomposition analysis was conducted using Stata MP 18.0 software. The level of statistical significance was set at 0.05.

### 2.10 Fairlie decomposition model

In this study, we employed the multivariate Fairlie Decomposition based on a binary logit model to quantitatively decompose the sources of gender disparities in eHealth literacy. FDA is a multivariate modeling technique designed to quantify the contribution of differences in two sets of predictors to the outcome variable. This method is an extension of Blinder-Oaxaca decomposition, which has been widely criticized for its inefficiency when dealing with logistic and probabilistic regression models. The FDA was developed specifically for nonlinear regression models, including logit and probit models (40). The FDA identifies the contribution of independent variables to explaining differences between groups by calculating the change in the mean predicted probability resulting from substituting one independent variable at a time in one group (e.g., Group A—male university students) while holding other variables constant in the other group (e.g., Group B—female university students). The Fairlie decomposition technique works by limiting the predicted probability to between 0 and 1. Studies have shown that the FDA can better quantify the contribution and significance levels of different variables for nonlinear regression models (10, 26, 41). Given that the dependent variable was a dichotomous variable, we employed Fairlie’s nonlinear decomposition to decompose the discrepancies in university students’ eHealth literacy into the contributions of various factors. As outlined by Fairlie (42), when male students are used as the reference group, the decomposition of the nonlinear equation can be expressed as follows:

$$\begin{array}{l} {\bar{Y}}_{m}-{\bar{Y}}_{f}=[\sum_{i=1}^{N^{m}}\frac{F(X_{i}^{m}\beta^{m})}{N^{m}}-\sum_{i=1}^{N^{f}}\frac{F(X_{i}^{f}\beta^{m})}{N^{f}}]+\\ {}[\sum_{i=1}^{N^{f}}\frac{F(X_{i}^{f}\beta^{m})}{N^{f}}-\sum_{i=1}^{N^{f}}\frac{F(X_{i}^{f}\beta^{f})}{N^{f}}]\;\; \end{array}$$ (1)

The average probabilities of the binary eHealth literacy outcomes for the male and female groups are denoted by ${\bar{Y}}_{m}$ and ${\bar{Y}}_{f}$ , respectively. The F is employed to indicate the cumulative distribution function of the logistic distribution. Ym-Yf represents the total difference in the differences between the male and female groups. The $N^{m}$ and $N^{f}$ represent the sample sizes of the male and female groups. The first term in parentheses in Equation (1) represents the portion of the gap attributable to differences in observed characteristics, while the second term denotes the portion of the gap due to differences in estimated coefficients.

For model implementation, all categorical covariates were entered into the decomposition model in the form of dummy variables. Variables were included in the model following a logical order from demographic characteristics, socioeconomic characteristics, lifestyle behaviors to mental health status. Bootstrap resampling with 100 replications was applied to estimate the standard errors and 95% confidence intervals of each decomposition term, to ensure the stability of the estimation results. We also performed a sensitivity test by adjusting the order of variable inclusion, and found that the contribution ranking and proportional size of each factor remained substantially unchanged, indicating that the decomposition results are robust and not sensitive to variable ordering.

## 3 Results

### 3.1 Basic characteristics of the study subjects

This study included a total of 7,230 samples. The average score of university students’ eHealth literacy was 29.22 ± 6.68, with male university students scoring an average of 29.51 ± 7.67 and female university students scoring 29.08 ± 6.11 (Table 1). An independent samples t-test revealed that male students had a significantly higher mean total score than female students (*t* = 2.58, df = 7,228, *p* = 0.010, Cohen’s *d* = 0.065). Previous studies on the eHealth literacy of Chinese university students have established a standard of 32 points (36), and those scoring below this threshold are considered to have not met the required standard. Results in Table 2 show that 4,135 (57.2%) university students did not meet the 32-point eHEALS cut-off, while 3,095 (42.8%) were qualified. Notably, the proportion of unqualified females (59.1%) was higher than that of males (53.4%; *p* < 0.001). The differences in religious belief and exercise between genders were not significant (*p* > 0.05).

**Table 1:** eHealth literacy score for university students.

Item	Overall (x ± s)	Male (x ± s)	Female (x ± s)
Q1: I know how to find helpful health resources on the Internet	3.69 ± 0.93	3.71 ± 1.05	3.68 ± 0.86
Q2: I know how to use the Internet to answer my health questions	3.67 ± 0.92	3.69 ± 1.05	3.67 ± 0.85
Q3: I know what health resources are available on the Internet	3.64 ± 0.91	3.68 ± 1.03	3.62 ± 0.84
Q4: I know where to find helpful health resources on the Internet	3.65 ± 0.90	3.67 ± 1.02	3.64 ± 0.84
Q5: I know how to use the health information I find on the Internet to help me	3.69 ± 0.89	3.71 ± 1.01	3.67 ± 0.83
Q6: I have the skills I need to evaluate the health resources I find on the Internet	3.63 ± 0.90	3.68 ± 1.02	3.61 ± 0.84
Q7: I can tell high quality from low quality health resources on the Internet	3.67 ± 0.90	3.72 ± 1.02	3.64 ± 0.83
Q8: I feel confident in using information from the Internet to make health decisions	3.59 ± 0.90	3.66 ± 1.01	3.55 ± 0.84
Total score	29.22 ± 6.68	29.51 ± 7.67	29.08 ± 6.11

**Table 2:** Distribution of the variables in male and female respondents.

Variable	Male [n(%)]	Female [n(%)]	*x^2^*	*p*
eHealth literacy			20.745	<0.001
≥32	1,122 (46.6)	1,973 (40.9)		
<32	1,288 (53.4)	2,847 (59.1)		
Type of household			58.356	<0.001
Urban	1,055 (43.8)	1,665 (34.5)		
Rural	1,355 (56.2)	3,155 (65.5)		
Religious belief			2.441	0.127
Yes	148 (6.1)	251 (5.2)		
No	2,262 (93.9)	4,569 (94.8)		
Class ranking			77.577	<0.001
<25.0%	590 (24.5)	1,408 (29.2)		
25.0%–<50.0%	822 (34.1)	1,827 (37.9)		
50.0%–<75.0%	677 (28.1)	1,214 (25.2)		
≥75.0%	321 (13.3)	371 (7.7)		
Monthly per capita household income			68.538	<0.001
<2,500RMB	392 (16.3)	985 (20.4)		
2,500–5,000RMB	914 (37.9)	2,101 (43.6)		
5,000–10,000RMB	787 (32.7)	1,195 (24.8)		
≥10,000RMB	317 (13.1)	539 (11.2)		
Exercise			0.027	0.870
Yes	1,695 (70.3)	3,399 (70.5)		
No	715 (29.7)	1,421 (29.5)		
Smoking			258.938	<0.001
Yes	187 (7.8)	38 (0.8)		
No	2,223 (92.2)	4,782 (99.2)		
Drinking			224.023	<0.001
Yes	613 (25.4)	562 (11.7)		
No	1,797 (74.6)	4,258 (88.3)		
GAD-7			47.487	<0.001
≥5	803 (33.3)	2,010 (41.7)		
<5	1,607 (66.7)	2,810 (58.3)		
PHQ-9			68.939	<0.001
≥5	955 (39.6)	2,408 (50.0)		
<5	1,455 (60.4)	2,412 (50.0)		

### 3.2 Comparison of variables’ distribution

Table 3 demonstrates the relationship between the distribution of covariates and qualified or unqualified eHealth literacy scores for male and female university students. The data indicate that certain covariate distributions exhibit similar characteristics among students who qualified or unqualified in the eHealth literacy assessment. These results reveal the differences in the composition of covariates between different genders within each literacy group, providing a foundation for subsequent analysis. The univariate variable screening for the multivariable logistic regression was based on the overall association between each covariate and eHealth literacy status in the full sample. Variables identified as statistically significant in univariate tests were consistent with the distribution patterns shown in Table 3, including type of household, class ranking, monthly per capita household income, smoking, drinking, GAD-7, and PHQ-9.

**Table 3:** Distribution of the variables in unqualified eHealth literacy and qualified eHealth literacy respondents.

Variable	Unqualified eHealth literacy				Qualified eHealth literacy			
	Male [n(%)]	Female [n(%)]	*x^2^*	*p*	Male [n(%)]	Female [n(%)]	*x^2^*	*p*
Type of household			30.794	<0.001			19.263	<0.001
Urban	481 (37.3)	817 (28.7)			574 (51.2)	848 (43.0)		
Rural	807 (62.7)	2,030 (71.3)			548 (48.8)	1,125 (57.0)		
Religious belief			1.620	0.212			0.849	0.370
Yes	80 (6.2)	149 (5.2)			68 (6.1)	104 (5.3)		
No	1,208 (93.8)	2,698 (94.8)			1,054 (93.9)	1,869 (94.7)		
Class ranking			36.763	<0.001			49.410	<0.001
<25.0%	293 (22.7)	756 (26.6)			297 (26.5)	652 (33.0)		
25.0%–<50.0%	432 (33.5)	1,087 (38.2)			390 (34.8)	740 (37.5)		
50.0%–<75.0%	381 (29.6)	751 (26.4)			296 (26.4)	463 (23.5)		
≥75.0%	182 (14.1)	253 (8.9)			139 (12.4)	118 (6.0)		
Monthly per capita household income			38.445	<0.001			24.963	<0.001
<2,500RMB	233 (18.1)	673 (23.6)			159 (14.2)	312 (15.8)		
2,500–5,000RMB	550 (42.7)	1,314 (46.2)			364 (32.4)	787 (39.9)		
5,000–10,000RMB	376 (29.2)	612 (21.5)			411 (36.6)	583 (29.5)		
≥10,000RMB	129 (10.0)	248 (8.7)			188 (16.8)	291 (14.7)		
Exercise			4.782	0.029			0.272	0.630
Yes	728 (56.5)	1,712 (60.1)			967 (86.2)	1,687 (85.5)		
No	560 (43.5)	1,135 (39.9)			155 (13.8)	286 (14.5)		
Smoking			173.525	<0.001			90.527	<0.001
Yes	114 (8.9)	25 (0.9)			73 (6.5)	13 (0.7)		
No	1,174 (91.1)	2,822 (99.1)			1,049 (93.5)	1,960 (99.3)		
Drinking			158.801	<0.001			70.176	<0.001
Yes	345 (26.8)	320 (11.2)			268 (23.9)	242 (12.3)		
No	943 (73.2)	2,527 (88.8)			854 (76.1)	1,731 (87.7)		
GAD-7			12.383	<0.001			32.010	<0.001
≥5	526 (40.8)	1,330 (46.7)			277 (24.7)	680 (34.5)		
<5	762 (59.2)	1,517 (53.3)			845 (75.3)	1,293 (65.5)		
PHQ-9			18.007	<0.001			46.050	<0.001
≥5	631 (49.0)	1,597 (56.1)			324 (28.9)	811 (41.1)		
<5	657 (51.0)	1,250 (43.9)			798 (71.1)	1,162 (58.9)		

### 3.3 Logistic model results

Table 4 presents the results of logistic modeling for eHealth literacy qualification among male and female university students. Among male university students, rural household registration (OR = 0.666) and lack of regular exercise (OR = 0.218) were associated with lower odds of achieving qualified eHealth literacy, whereas monthly per capita household income≥10,000RMB (OR = 1.494) and absence of depressive symptoms (PHQ-9 < 5, OR = 1.945) were associated with higher odds of achieving qualified eHealth literacy. Among female university students, rural household registration (OR = 0.642), lower class rankings (25.0 to <50.0%, OR = 0.791; 50.0 to <75.0%, OR = 0.797; ≥75.0%, OR = 0.704), and lack of regular exercise (OR = 0.289) were associated with lower odds of achieving qualified eHealth literacy. Monthly per capita household income of 5,000–10,000RMB (OR = 1.598) and ≥10,000RMB (OR = 1.690), and absence of depressive symptoms (PHQ-9 < 5, OR = 1.454) were associated with higher odds of achieving qualified eHealth literacy.

**Table 4:** Logistic regression results for factors associated with qualified eHealth literacy (eHEALS ≥32) among male and female university students.

Variable	Overall		Male		Female	
	OR	95%CI	OR	95%CI	OR	95%CI
Type of household						
Urban (reference)	1		1		1	
Rural	0.641^c^	(0.574,0.715)	0.666^c^	(0.552,0.803)	0.642^c^	(0.560,0.736)
Religious belief						
Yes (reference)	1		1		1	
No	0.894	(0.718,1.112)	0.890	(0.616,1.286)	0.903	(0.687,1.187)
Class ranking						
<25.0% (reference)	1		1		1	
25.0%–<50.0%	0.816^b^	(0.720,0.925)	0.878	(0.697,1.107)	0.791^b^	(0.681,0.918)
50.0%–<75.0%	0.811^b^	(0.708,0.930)	0.829	(0.650,1.058)	0.797^b^	(0.675,0.941)
≥75.0%	0.806^a^	(0.666,0.976)	0.908	(0.672,1.227)	0.704^b^	(0.543,0.911)
Monthly per capita household income						
<2,500RMB (reference)	1		1		1	
2,500–5,000RMB	1.026	(0.890,1.183)	0.800	(0.615,1.040)	1.133	(0.956,1.343)
5,000–10,000RMB	1.491^c^	(1.276,1.743)	1.216	(0.925,1.598)	1.598^c^	(1.319,1.936)
≥10,000RMB	1.645^c^	(1.353,2.000)	1.494^a^	(1.060,2.106)	1.690^c^	(1.330,2.147)
Exercise						
Yes (reference)	1		1		1	
No	0.265^c^	(0.235,0.299)	0.218^c^	(0.177,0.269)	0.289^c^	(0.248,0.335)
Smoking						
Yes (reference)	1		1		1	
No	1.111	(0.818,1.511)	1.187	(0.832,1.693)	1.108	(0.534,2.300)
Drinking						
Yes (reference)	1		1		1	
No	0.963	(0.836,1.110)	1.107	(0.896,1.368)	0.899	(0.739,1.093)
GAD-7						
≥5 (reference)	1		1		1	
<5	1.150	(0.993,1.332)	1.177	(0.890,1.556)	1.133	(0.952,1.347)
PHQ-9						
≥5 (reference)	1		1		1	
<5	1.607^c^	(1.392,1.854)	1.945^c^	(1.487,2.545)	1.454^c^	(1.226,1.724)

a Significant at *p <* 0.05.

b Significant at *p <* 0.01.

c Significant at *p <* 0.001.

In summary, the disparities in the factors associated with qualified eHealth literacy between male and female university students were mainly reflected in class ranking. For female university students, class ranking showed a statistically significant association with qualified eHealth literacy, whereas no such association was observed for male university students.

### 3.4 Decomposition analysis results

Bootstrap resampling with 100 replications was used to estimate the standard errors and 95% confidence intervals of the decomposition estimates to ensure the stability of the results. Table 5 presents the results of the decomposition model showing the disparities in eHealth literacy levels between male and female university students. The results indicate that 45.0% of the observed factors contributed to the differences in eHealth literacy levels, while 55.0% can be attributed to unmeasured variables, differences in coefficients, measurement error, or residual confounding. Significant contributing factors (*p* < 0.001) were PHQ-9 (28.57%), type of household (14.29%), monthly per capita household income (10.71%), and exercise (−3.57%).

**Table 5:** The Fairlie decomposition model of eHealth literacy status in male and female university students.

Terms of decomposition	eHealth literacy status
Difference	0.056
Explained (%)	0.0252 (45.0)
Unexplained (%)	0.0308 (55.0)

Contribution to differences	*β*	*p*	Contribution (%)	[95%CI]
Explained				
PHQ-9	0.016	<0.001	28.57	(0.009,0.023)
Type of household	0.008	<0.001	14.29	(0.004,0.011)
Monthly per capita household income	0.006	0.001	10.71	(0.003,0.010)
GAD-7	0.003	0.255	5.36	(−0.003,0.009)
Religious belief	0.0002	0.572	0.36	(−0.0005,0.0009)
Class ranking	−0.001	0.485	−1.79	(−0.005,0.002)
Smoking	−0.002	0.341	−3.57	(−0.007,0.002)
Exercise	−0.002	<0.001	−3.57	(−0.003,-0.001)
Drinking	−0.003	0.343	−5.36	(−0.009,0.003)

## 4 Discussion

Based on cross-sectional data from 7,230 Chinese university students, this study explored the characteristics of gender disparities in eHealth literacy among university students. Using binary logistic regression and the Fairlie decomposition model, it identified key factors contributing to the disparity and the contribution of each factor. The results indicate that, influenced by multiple factors, there is a significant disparity in eHealth literacy levels between male and female university students. This disparity is jointly affected by observable factors such as depressive symptoms, type of household, family income, as well as an unexplained component encompassing unmeasured confounders, coefficient differences, and residual variation.

This study found that the average eHealth literacy score for Chinese university students was 29.22 ± 6.68, with 57.2% below the 32-point eHEALS cut-off, consistent with prior research (24). The average score for male university students (29.51 ± 7.67) was significantly higher than that for female students (29.08 ± 6.11; *t* = 2.58, df = 7,228, *p* = 0.010, Cohen’s *d* = 0.065), and the proportion of males meeting the 32-point eHEALS cut-off (46.6%) was also significantly higher than that of females (40.9%; *x^2^* = 20.745, *p* < 0.001). This finding is consistent with some international studies (43–45). Research by Deursen et al. pointed out that males often exhibit higher self-efficacy of control in activities involving technology use, which could potentially extend to the processes of seeking and evaluating health information (46). Another study among adults also found that men had greater confidence in using digital tools to solve health problems (47). This disparity may stem from the shaping of gender roles by social culture, leading to different roles and statuses for men and women in physiological, social, and family contexts, consequently affecting their opportunities for internet access (48). However, other studies have presented different conclusions. Some scholars found no significant gender disparities in eHealth literacy (49–51); Giger et al.’s survey among rural adolescents in the United States also did not find significant gender disparities in eHealth literacy (52). These varying results regarding gender and eHealth literacy may be influenced by cultural background, sample characteristics, or the specific dimensions of eHealth literacy assessed, suggesting that the issue has a certain degree of contextual dependence (53).

By constructing logistic regression models, this study identified key factors associated with eHealth literacy for male and female university students separately. This analysis revealed that gender disparities are not only evident in average levels but are also deeply reflected in the composition and effect sizes of influencing factors, providing empirical evidence for understanding the specific pathways through which disparities form. Despite the gender disparities in eHealth literacy among university students, male and female student groups share important commonalities in the factors associated with eHealth literacy, offering potential intervention points for improving the overall eHealth literacy level of this population.

Regular exercise was consistently associated with higher odds of achieving qualified eHealth literacy in both groups. Among male (OR = 0.218) and female (OR = 0.289) university students, those without regular exercise habits were significantly less likely to achieve adequate eHealth literacy compared to those with exercise habits. This finding is highly consistent with other research conclusions (16). It is worth noting that exercise habits showed almost identical distributions across genders (*p* = 0.870), indicating that the strong association between exercise and eHealth literacy reflects a general health-promoting pattern applicable to both genders rather than a gender-specific mechanism. A possible explanation is that the process of exercising itself can serve as a trigger for seeking health information; individuals may actively learn about nutrition and exercise-related knowledge to improve their performance, thereby enhancing their ability to use eHealth information in practice. Conversely, students lacking exercise habits may have weaker needs for health information, and their related skills may not be effectively developed. Therefore, this study suggests that advocating for and building a supportive environment to promote regular exercise among university students is one of the foundational strategies for enhancing their eHealth literacy. The absence of depressive symptoms was associated with higher odds of achieving qualified eHealth literacy among both male and female university students (male OR = 1.945, female OR = 1.454, *p* < 0.001). Depressive symptoms are often accompanied by cognitive impairments, such as difficulty concentrating, decreased decision-making and executive function, and slower information processing speed (54). Such cognitive impairments may affect an individual’s ability to seek, understand, and evaluate complex online health information. Simultaneously, feelings of worthlessness and low self-efficacy associated with depressive symptoms have been shown to correlate with reduced proactive health information seeking (55). Therefore, this study considers it highly important and necessary to effectively integrate mental health services, particularly depression screening and intervention, into health literacy promotion efforts for university students.

The results also showed that rural household registration was associated with lower odds of achieving qualified eHealth literacy among both male and female students (male OR = 0.666, female OR = 0.642, *p* < 0.001). This aligns with the conclusions of most previous studies (26, 56, 57). The impact of uneven resource distribution between urban and rural areas on health literacy is universal. This may be because university students living in urban areas have earlier access to and experience with the internet, while rural areas face disadvantages in electronic device accessibility, internet utilization, and health information dissemination channels, directly limiting this group’s ability to access and utilize online health resources. Therefore, efforts should be strengthened to enhance network resource construction in rural areas, creating more opportunities for rural students to access and apply health information online. Logistic regression analysis also revealed gender heterogeneity in influencing factors. These factors are crucial for constituting and explaining gender disparities and form the core basis for developing differentiated intervention strategies. Class ranking was a risk factor specific to female university students. Compared to female students ranking in the top 25%, those with lower rankings (especially in the ≥75% percentile) had significantly lower rates of achieving adequate literacy (OR = 0.704), whereas the effect of class ranking was not statistically significant among males (*p* > 0.05). This may reflect that women’s ability to access and utilize health information is more dependent on educational attainment and academic self-confidence. This result also aligns with findings from some studies indicating that women are more susceptible to the influence of self-efficacy during the information evaluation stage (55).

Through the Fairlie decomposition model, this study systematically quantified the contribution of observed and unobserved factors to the gender disparities in eHealth literacy among the sampled Chinese university students, providing empirical evidence for a deeper understanding of the mechanisms underlying eHealth literacy inequality from a gender perspective. The results showed that 45.0% of the disparity in eHealth literacy between genders could be attributed to observed factors, including PHQ-9 score (28.57%), type of household (14.29%), monthly per capita household income (10.71%), and exercise (−3.57%). The remaining 55.0% was attributed to unmeasured variables, differences in coefficients, measurement error, or residual confounding. This result not only confirms the complexity of gender disparities but also provides a clear quantitative basis for understanding their composition.

Within the explained portion, depressive symptoms (PHQ-9) were the single largest contributing factor, explaining 28.57% of the total disparity. This finding highlights the close association between mental health status and eHealth literacy performance. Depressive symptoms may be related to impaired cognitive function, reduced information-seeking motivation, and lower self-efficacy, which could in turn correlate with poorer capacity to search, evaluate, and apply online health information (17, 58, 59). The analysis results in Table 2 show that the proportion of female university students with depressive symptoms (PHQ-9 ≥ 5) was 50.0%, higher than that of males. The higher prevalence of depressive symptoms among female students in our sample may be associated with their lower eHealth literacy, which may be related to cognitive impairments or reduced self-efficacy associated with depression (60). Given the cross-sectional study design, the direction of this association needs further clarification. Combined with the logistic regression results, the absence of depressive symptoms was a protective factor for achieving adequate eHealth literacy, and this protective effect was stronger for males, suggesting that the association between depressive symptoms and lower eHealth literacy may be particularly pronounced among females. The two socioeconomic factors, type of household and monthly per capita household income, together contributed approximately 25% of the gender disparity in this study. Rural environmental background and lower family income constitute a dual disadvantage for accessing electronic information resources, and these factors may have a more pronounced impact on female students. Traditional gender role perceptions in resource-limited families may lead to implicit gender biases in investments related to electronic devices and education, resulting in females facing more restrictions in accessing digital devices and using the internet. This can lead to a lower starting point in digital skills (61, 62). This finding provides empirical evidence for considering the range of gender-related impacts when formulating policies aimed at promoting educational equity and digital inclusion, ensuring that resources and support effectively reach disadvantaged female university students. The contribution of exercise to the gender disparities was negative (−3.57%). This may be because exercise habits exhibit high gender homogeneity, and its positive impact on eHealth literacy is relatively balanced across male and female student groups, thereby partially offsetting the gender gap. This does not diminish the importance of exercise as a factor in promoting health. On the contrary, it reflects that exercise habits are equally prevalent among both genders and can bring similar benefits to both.

However, 55.0% of the gender disparities remained unexplained by the measured variables in this study. This unexplained portion profoundly reveals the complexity of the causes of the gender gap. It may stem from multiple deeper causes not captured by our measurements. Firstly, this result may reflect disparities in intrinsic traits related to gender itself, such as variations in health information-seeking motivation, risk preferences, or cognitive processing. Some research suggests individuals differ in their criteria for evaluating online health information (63), and this factor may manifest differently across genders. Second, long-standing stereotypes regarding gender roles within the socio-cultural environment subtly influence the self-efficacy of different gender groups, their interest in the health domain, and their confidence in using digital resources to solve problems. These difficult-to-quantify socio-psychological factors constitute an important background for the observed disparities. Relevant research confirms significant gender disparities in the acquisition and usage patterns of digital skills (46), which may be closely related to gender concepts formed during the socialization process. Amoah et al., studying the relationship between health literacy and socio-demographic and behavioral factors from a gender perspective among residents in a Ghanaian district, found that deeply ingrained cultural gender roles profoundly influence health information behavior. Women are often assigned the traditional role of family caregivers, and although they may come into contact with health information more frequently, this contact is often limited to superficial content like daily care, lacking systematic training in information filtering, evaluation, and application. Men, on the other hand, are more likely to receive social encouragement in exploring digital resources and solving problems, forming a more proactive pattern of information acquisition (62). These unexplained gender disparities largely reflect the influence of multiple deep-seated factors, including social culture, individual characteristics, and inherent gender cognitive patterns. These factors are difficult to capture through traditional quantitative indicators but can have a persistent impact on the formation of eHealth literacy. This necessitates that relevant strategies incorporate a gender perspective, addressing not only visible resource and health inequalities but also paying attention to less visible gender disparities in aspects like cognition and motivation.

Based on the above findings, we propose several potential intervention directions that may be considered for narrowing the gender gap in eHealth literacy among university students. First, it is important to focus on and address the disadvantages faced by female university students arising from inequalities caused by socio-cultural and economic factors (11). Fostering a positive social atmosphere of gender equality and building a socio-cultural environment that respects diverse differences may help reduce gender-related constraints on digital health capability development. Universities could consider enhancing the understanding of gender equality among faculty and students through activities like gender equality education lectures and thematic seminars, which might help to reduce the constraints of gender stereotypes on female university students’ engagement with eHealth information. Improving the accessibility of eHealth resources is an important approach to narrow the gender gap. The government and universities can collaborate to provide support such as electronic device subsidies and free data packages for students from economically disadvantaged areas (especially female students), reducing the cost of accessing health information and creating a relatively equitable digital network environment (64). Relevant research indicates that targeted health promotion interventions can significantly improve the self-efficacy of resource-poor groups in the digital health environment and promote their health behaviors (65). Secondly, as the primary settings for students’ academic and daily life, universities may consider further strengthening campus mental health support systems. Establishing routine mental health screening covering all students, paying attention to the mental health status of female students, and providing early psychological support may help improve students’ cognitive function and information processing capacity. By establishing online psychological counseling platforms, providing group psychological counseling, and offering cognitive-behavioral intervention courses for depression, universities can improve students’ cognitive function and information processing capabilities (60, 66). Universities can also integrate mental health education into training programs aimed at enhancing eHealth literacy, helping students at high risk of depression rebuild their self-efficacy in information seeking (59). In addition, universities can cultivate an active and vibrant campus sports culture. Adding and upgrading sports facilities, enriching sports clubs and activities, and integrating exercise into campus life may help stimulate students’ intrinsic motivation to engage in physical activity and further promote their attention to health information. Exercise not only directly benefits health but also creates opportunities for students to learn health knowledge, prompting them to actively seek health information related to nutrition, injury prevention, etc., thereby enhancing their eHealth literacy through practice.

In summary, narrowing the gender gap in eHealth literacy among Chinese university students and improving the overall level requires collaborative efforts from the government, universities, and various sectors of society. Through multi-dimensional and targeted intervention strategies, barriers hindering the improvement of female university students’ eHealth literacy can be effectively removed, promoting the balanced development of eHealth literacy among all university students.

## 5 Limitations and strengths

This study explored gender disparities in eHealth literacy among Chinese university students and utilized the Fairlie decomposition model to quantify the sources of these disparities. However, this study also has the following limitations: First, as a cross-sectional questionnaire survey, it cannot establish causal relationships between variables. In particular, the association between depressive symptoms and lower eHealth literacy may be bidirectional. Depressive symptoms may impair cognitive function and reduce health information seeking, while poor eHealth literacy may lead to increased health anxiety and subsequent depressive symptoms. Longitudinal studies are needed to clarify the causal direction. Second, the data were derived from self-reports, which may be subject to social desirability bias or recall bias. Third, although the sample covered multiple regions with a large sample size, the convenience sampling method may introduce selection bias, and the number of female samples is twice that of male samples. Therefore, this may limit the universality of the research results to all Chinese university students. Fourth, the use of online surveys may also lead to selection bias. Since eHealth literacy is intrinsically linked to internet access and digital literacy, students with better digital access or a stronger interest in health information are more likely to participate in online surveys, which may result in a certain degree of overestimation of the overall level of eHealth literacy in the sample. Fifth, despite considering various factors, it is still possible that other variables that could contribute to explaining the remaining disparity were omitted, such as academic major, frequency of internet use, ownership of digital devices, previous health education exposure and other relevant factors. The absence of these variables may lead to residual confounding.

## 6 Conclusion

Through regression analysis and the Fairlie decomposition model, this study revealed a significant gender disparity in the eHealth literacy of the sampled Chinese university students, with male students exhibiting higher eHealth literacy levels than female students. Of this gender disparity, 45.0% was explained by observable factors, among which depressive symptoms (PHQ-9) contributed the most (28.57%), followed by type of household (14.29%) and monthly per capita household income (10.71%), while exercise level showed a negative contribution (−3.57%).

Based on the study findings, the following potential policy and practice directions are proposed to address the gender disparities in eHealth literacy: (1) Fostering a social atmosphere of gender equality to help reduce the constraints of gender stereotypes on female university students’ access to eHealth information. (2) Optimizing digital resource allocation and providing targeted support for students from rural and low-income families, to lower the threshold for accessing online health information. (3) Strengthening campus mental health services and carrying out routine depression screening and supportive interventions, which may help improve students’ cognitive function and information processing capacity. (4) Building a positive campus sports culture to stimulate students’ motivation to seek and apply health information.

This study provides new insights into the gender disparities in eHealth literacy among Chinese university students and offers empirical evidence and a theoretical basis for formulating and improving policies aimed at enhancing eHealth literacy.

## Data Availability

The original contributions presented in the study are included in the article/Supplementary material, further inquiries can be directed to the corresponding authors.

## Footnotes

1
https://www.wjx.cn/
