# Supplementary material for: Gender disparities in eHealth literacy among Chinese university students and their decomposition
Source: Front Public Health. 2026 Sep 3;14:1919426. doi: 10.3389/fpubh.2026.1919426 (PMC13582514; doi:10.3389/fpubh.2026.1919426)
Supplement: Supplementary file 1 [file Table_1.DOCX]

**Gender disparities in eHealth literacy among**

**Chinese university students and their decomposition**

**Supplementary files**

**Table**

**Table 1.** eHealth literacy score for university students

| Item | OVERALL  (x±s) | MALE  (x±s) | FEMALE  (x±s) |
| --- | --- | --- | --- |
| Q1: I know how to find helpful health resources on the Internet | 3.69±0.93 | 3.71±1.05 | 3.68±0.86 |
| Q2: I know how to use the Internet to answer my health questions | 3.67±0.92 | 3.69±1.05 | 3.67±0.85 |
| Q3: I know what health resources are available on the Internet | 3.64±0.91 | 3.68±1.03 | 3.62±0.84 |
| Q4: I know where to find helpful health resources on the Internet | 3.65±0.90 | 3.67±1.02 | 3.64±0.84 |
| Q5: I know how to use the health information I find on the Internet to help me | 3.69±0.89 | 3.71±1.01 | 3.67±0.83 |
| Q6: I have the skills I need to evaluate the health resources I find on the Internet | 3.63±0.90 | 3.68±1.02 | 3.61±0.84 |
| Q7: I can tell high quality from low quality health resources on the Internet | 3.67±0.90 | 3.72±1.02 | 3.64±0.83 |
| Q8: I feel confident in using information from the Internet to make health decisions | 3.59±0.90 | 3.66±1.01 | 3.55±0.84 |
| Total score | 29.22±6.68 | 29.51±7.67 | 29.08±6.11 |

**Table 2.** Distribution of the variables in male and female respondents

| VARIABLE | MALE[n(%)] | FEMALE[n(%)] | *x^2^* | *P* |
| --- | --- | --- | --- | --- |
| **eHealth literacy** |  |  | 20.745 | <0.001 |
| ≥32 | 1122 (46.6) | 1973 (40.9) |  |  |
| <32 | 1288 (53.4) | 2847 (59.1) |  |  |
| **Type of household** |  |  | 58.356 | <0.001 |
| Urban | 1055 (43.8) | 1665 (34.5) |  |  |
| Rural | 1355 (56.2) | 3155 (65.5) |  |  |
| **Religious belief** |  |  | 2.441 | 0.127 |
| Yes | 148 (6.1) | 251 (5.2) |  |  |
| No | 2262 (93.9) | 4569 (94.8) |  |  |
| **Class ranking** |  |  | 77.577 | <0.001 |
| <25.0% | 590 (24.5) | 1408 (29.2) |  |  |
| 25.0%~<50.0% | 822 (34.1) | 1827 (37.9) |  |  |
| 50.0%~<75.0% | 677 (28.1) | 1214 (25.2) |  |  |
| ≥75.0% | 321 (13.3) | 371 (7.7) |  |  |
| **Monthly per capita household income** |  |  | 68.538 | <0.001 |
| <2,500RMB | 392 (16.3) | 985 (20.4) |  |  |
| 2,500~5,000RMB | 914 (37.9) | 2101 (43.6) |  |  |
| 5,000~10,000RMB | 787 (32.7) | 1195 (24.8) |  |  |
| ≥10,000RMB | 317 (13.1) | 539 (11.2) |  |  |
| **Exercise** |  |  | 0.027 | 0.870 |
| Yes | 1695 (70.3) | 3399 (70.5) |  |  |
| No | 715 (29.7) | 1421 (29.5) |  |  |
| **Smoking** |  |  | 258.938 | <0.001 |
| Yes | 187 (7.8) | 38 (0.8) |  |  |
| No | 2223 (92.2) | 4782 (99.2) |  |  |
| **Drinking** |  |  | 224.023 | <0.001 |
| Yes | 613 (25.4) | 562 (11.7) |  |  |
| No | 1797 (74.6) | 4258 (88.3) |  |  |
| **GAD-7** |  |  | 47.487 | <0.001 |
| ≥5 | 803 (33.3) | 2010 (41.7) |  |  |
| <5 | 1607 (66.7) | 2810 (58.3) |  |  |
| **PHQ-9** |  |  | 68.939 | <0.001 |
| ≥5 | 955 (39.6) | 2408 (50.0) |  |  |
| <5 | 1455 (60.4) | 2412 (50.0) |  |  |

**Table 3.** Distribution of the variables in Unqualified eHealth literacy and Qualified eHealth literacy respondents

| VARIABLE | Unqualified eHealth literacy | | | | Qualified eHealth literacy | | | |
| --- | --- | --- | --- | --- | --- | --- | --- | --- |
|  | MALE  [n(%)] | FEMALE  [n(%)] | *x^2^* | *P* | MALE  [n(%)] | FEMALE  [n(%)] | *x^2^* | *P* |
| **Type of household** |  |  | 30.794 | <0.001 |  |  | 19.263 | <0.001 |
| Urban | 481 (37.3) | 817 (28.7) |  |  | 574 (51.2) | 848 (43.0) |  |  |
| Rural | 807 (62.7) | 2030 (71.3) |  |  | 548 (48.8) | 1125 (57.0) |  |  |
| **Religious belief** |  |  | 1.620 | 0.212 |  |  | 0.849 | 0.370 |
| Yes | 80 (6.2) | 149 (5.2) |  |  | 68 (6.1) | 104 (5.3) |  |  |
| No | 1208 (93.8) | 2698 (94.8) |  |  | 1054 (93.9) | 1869 (94.7) |  |  |
| **Class ranking** |  |  | 36.763 | <0.001 |  |  | 49.410 | <0.001 |
| <25.0% | 293 (22.7) | 756 (26.6) |  |  | 297 (26.5) | 652 (33.0) |  |  |
| 25.0%~<50.0% | 432 (33.5) | 1087 (38.2) |  |  | 390 (34.8) | 740 (37.5) |  |  |
| 50.0%~<75.0% | 381 (29.6) | 751 (26.4) |  |  | 296 (26.4) | 463 (23.5) |  |  |
| ≥75.0% | 182 (14.1) | 253 (8.9) |  |  | 139 (12.4) | 118 (6.0) |  |  |
| **Monthly per capita household income** |  |  | 38.445 | <0.001 |  |  | 24.963 | <0.001 |
| <2,500RMB | 233 (18.1) | 673 (23.6) |  |  | 159 (14.2) | 312 (15.8) |  |  |
| 2,500~5,000RMB | 550 (42.7) | 1314 (46.2) |  |  | 364 (32.4) | 787 (39.9) |  |  |
| 5,000~10,000RMB | 376 (29.2) | 612 (21.5) |  |  | 411 (36.6) | 583 (29.5) |  |  |
| ≥10,000RMB | 129 (10.0) | 248 (8.7) |  |  | 188 (16.8) | 291 (14.7) |  |  |
| **Exercise** |  |  | 4.782 | 0.029 |  |  | 0.272 | 0.630 |
| Yes | 728 (56.5) | 1712 (60.1) |  |  | 967 (86.2) | 1687 (85.5) |  |  |
| No | 560 (43.5) | 1135 (39.9) |  |  | 155 (13.8) | 286 (14.5) |  |  |
| **Smoking** |  |  | 173.525 | <0.001 |  |  | 90.527 | <0.001 |
| Yes | 114 (8.9) | 25 (0.9) |  |  | 73 (6.5) | 13 (0.7) |  |  |
| No | 1174 (91.1) | 2822 (99.1) |  |  | 1049 (93.5) | 1960 (99.3) |  |  |
| **Drinking** |  |  | 158.801 | <0.001 |  |  | 70.176 | <0.001 |
| Yes | 345 (26.8) | 320 (11.2) |  |  | 268 (23.9) | 242 (12.3) |  |  |
| No | 943 (73.2) | 2527 (88.8) |  |  | 854 (76.1) | 1731 (87.7) |  |  |
| **GAD-7** |  |  | 12.383 | <0.001 |  |  | 32.010 | <0.001 |
| ≥5 | 526 (40.8) | 1330 (46.7) |  |  | 277 (24.7) | 680 (34.5) |  |  |
| <5 | 762 (59.2) | 1517 (53.3) |  |  | 845 (75.3) | 1293 (65.5) |  |  |
| **PHQ-9** |  |  | 18.007 | <0.001 |  |  | 46.050 | <0.001 |
| ≥5 | 631 (49.0) | 1597 (56.1) |  |  | 324 (28.9) | 811 (41.1) |  |  |
| <5 | 657 (51.0) | 1250 (43.9) |  |  | 798 (71.1) | 1162 (58.9) |  |  |

**Table 4.** Logistic regression results for factors associated with qualified eHealth literacy (eHEALS ≥32) among male and female university students

| VARIABLE | Overall | | MALE | | FEMALE | |
| --- | --- | --- | --- | --- | --- | --- |
|  | OR | 95%CI | OR | 95%CI | OR | 95%CI |
| **Type of household** |  |  |  |  |  |  |
| Urban (reference) | 1 |  | 1 |  | 1 |  |
| Rural | 0.641^c^ | (0.574,0.715) | 0.666^c^ | (0.552,0.803) | 0.642^c^ | (0.560,0.736) |
| **Religious belief** |  |  |  |  |  |  |
| Yes (reference) | 1 |  | 1 |  | 1 |  |
| No | 0.894 | (0.718,1.112) | 0.890 | (0.616,1.286) | 0.903 | (0.687,1.187) |
| **Class ranking** |  |  |  |  |  |  |
| <25.0% (reference) | 1 |  | 1 |  | 1 |  |
| 25.0%~<50.0% | 0.816^b^ | (0.720,0.925) | 0.878 | (0.697,1.107) | 0.791^b^ | (0.681,0.918) |
| 50.0%~<75.0% | 0.811^b^ | (0.708,0.930) | 0.829 | (0.650,1.058) | 0.797^b^ | (0.675,0.941) |
| ≥75.0% | 0.806^a^ | (0.666,0.976) | 0.908 | (0.672,1.227) | 0.704^b^ | (0.543,0.911) |
| **Monthly per capita household income** |  |  |  |  |  |  |
| <2,500RMB (reference) | 1 |  | 1 |  | 1 |  |
| 2,500~5,000RMB | 1.026 | (0.890,1.183) | 0.800 | (0.615,1.040) | 1.133 | (0.956,1.343) |
| 5,000~10,000RMB | 1.491^c^ | (1.276,1.743) | 1.216 | (0.925,1.598) | 1.598^c^ | (1.319,1.936) |
| ≥10,000RMB | 1.645^c^ | (1.353,2.000) | 1.494^a^ | (1.060,2.106) | 1.690^c^ | (1.330,2.147) |
| **Exercise** |  |  |  |  |  |  |
| Yes (reference) | 1 |  | 1 |  | 1 |  |
| No | 0.265^c^ | (0.235,0.299) | 0.218^c^ | (0.177,0.269) | 0.289^c^ | (0.248,0.335) |
| **Smoking** |  |  |  |  |  |  |
| Yes (reference) | 1 |  | 1 |  | 1 |  |
| No | 1.111 | (0.818,1.511) | 1.187 | (0.832,1.693) | 1.108 | (0.534,2.300) |
| **Drinking** |  |  |  |  |  |  |
| Yes (reference) | 1 |  | 1 |  | 1 |  |
| No | 0.963 | (0.836,1.110) | 1.107 | (0.896,1.368) | 0.899 | (0.739,1.093) |
| **GAD-7** |  |  |  |  |  |  |
| ≥5 (reference) | 1 |  | 1 |  | 1 |  |
| <5 | 1.150 | (0.993,1.332) | 1.177 | (0.890,1.556) | 1.133 | (0.952,1.347) |
| **PHQ-9** |  |  |  |  |  |  |
| ≥5 (reference) | 1 |  | 1 |  | 1 |  |
| <5 | 1.607^c^ | (1.392,1.854) | 1.945^c^ | (1.487,2.545) | 1.454^c^ | (1.226,1.724) |

^a^*Significant at P < .05;*

^b^*Significant at P < .01.*

^c^*Significant at P < .001.*

**Table 5.** The Fairlie decomposition model of eHealth literacy status in male and female university students

| **Terms of decomposition** | | | **eHealth literacy status** | |
| --- | --- | --- | --- | --- |
| Difference | | | 0.056 | |
| Explained (%) | | | 0.0252 (45.0) | |
| Unexplained (%) | | | 0.0308 (55.0) | |
| **Contribution to differences** | ***β*** | ***P*** | **Contribution (%)** | **[95%CI]** |
| **Explained** |  |  |  |  |
| PHQ-9 | 0.016 | <0.001 | 28.57 | (0.009,0.023) |
| Type of household | 0.008 | <0.001 | 14.29 | (0.004,0.011) |
| Monthly per capita household income | 0.006 | 0.001 | 10.71 | (0.003,0.010) |
| GAD-7 | 0.003 | 0.255 | 5.36 | (-0.003,0.009) |
| Religious belief | 0.0002 | 0.572 | 0.36 | (-0.0005,0.0009) |
| Class ranking | -0.001 | 0.485 | -1.79 | (-0.005,0.002) |
| Smoking | -0.002 | 0.341 | -3.57 | (-0.007,0.002) |
| Exercise | -0.002 | <0.001 | -3.57 | (-0.003,-0.001) |
| Drinking | -0.003 | 0.343 | -5.36 | (-0.009,0.003) |
